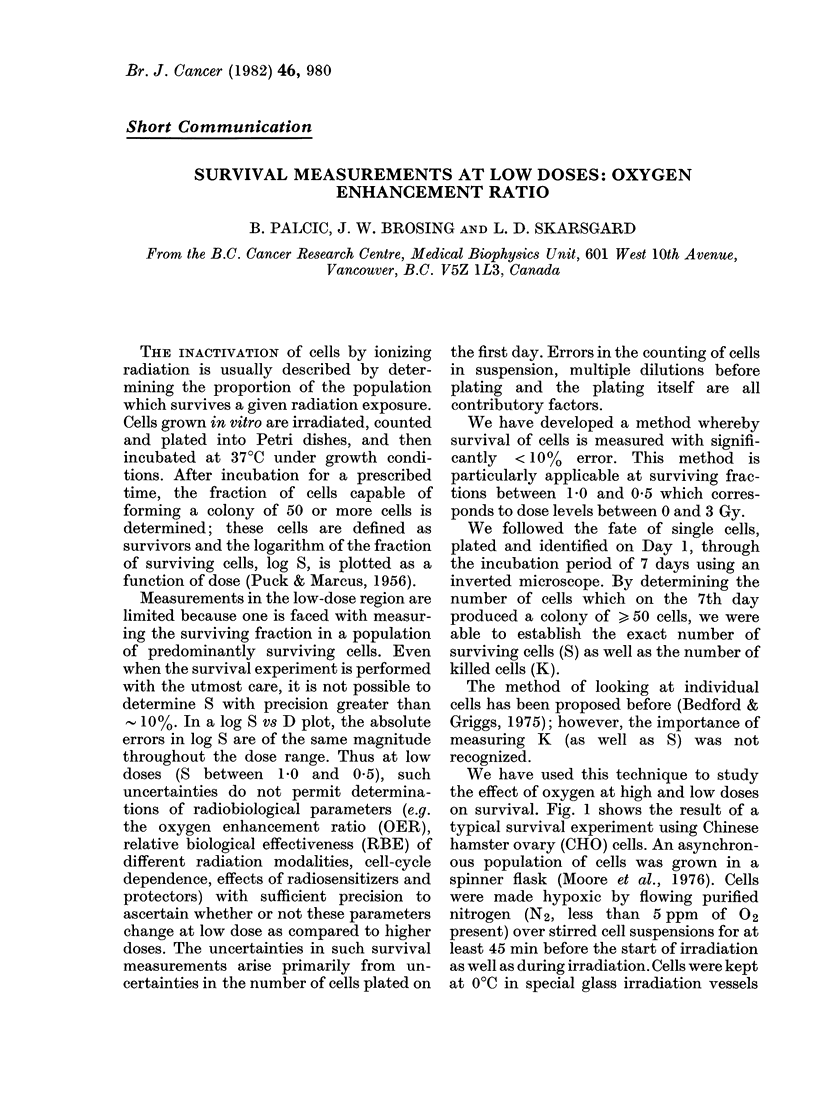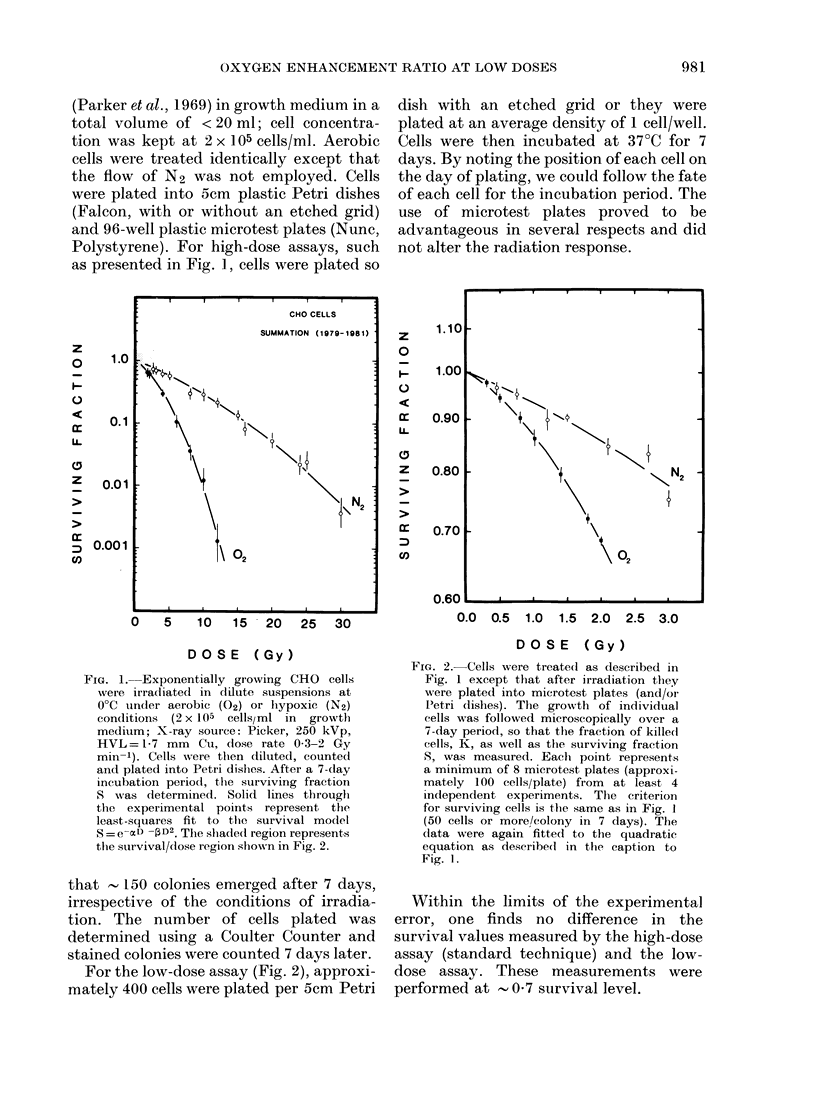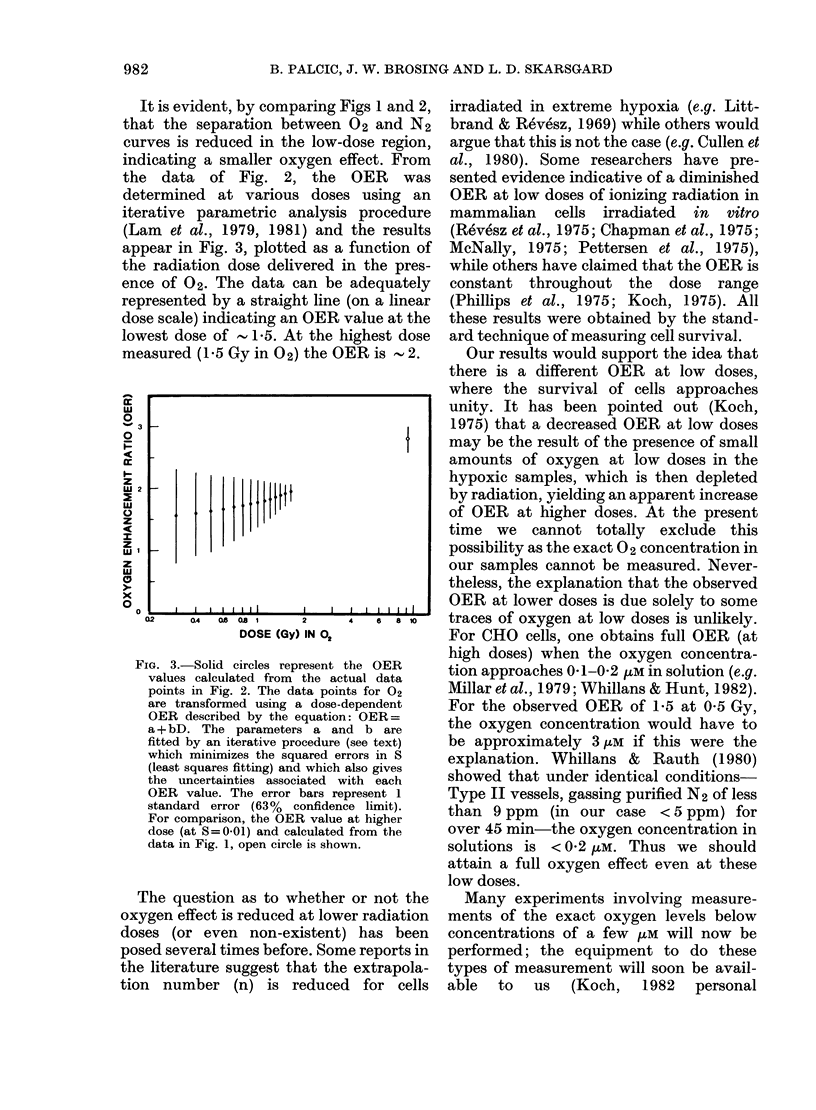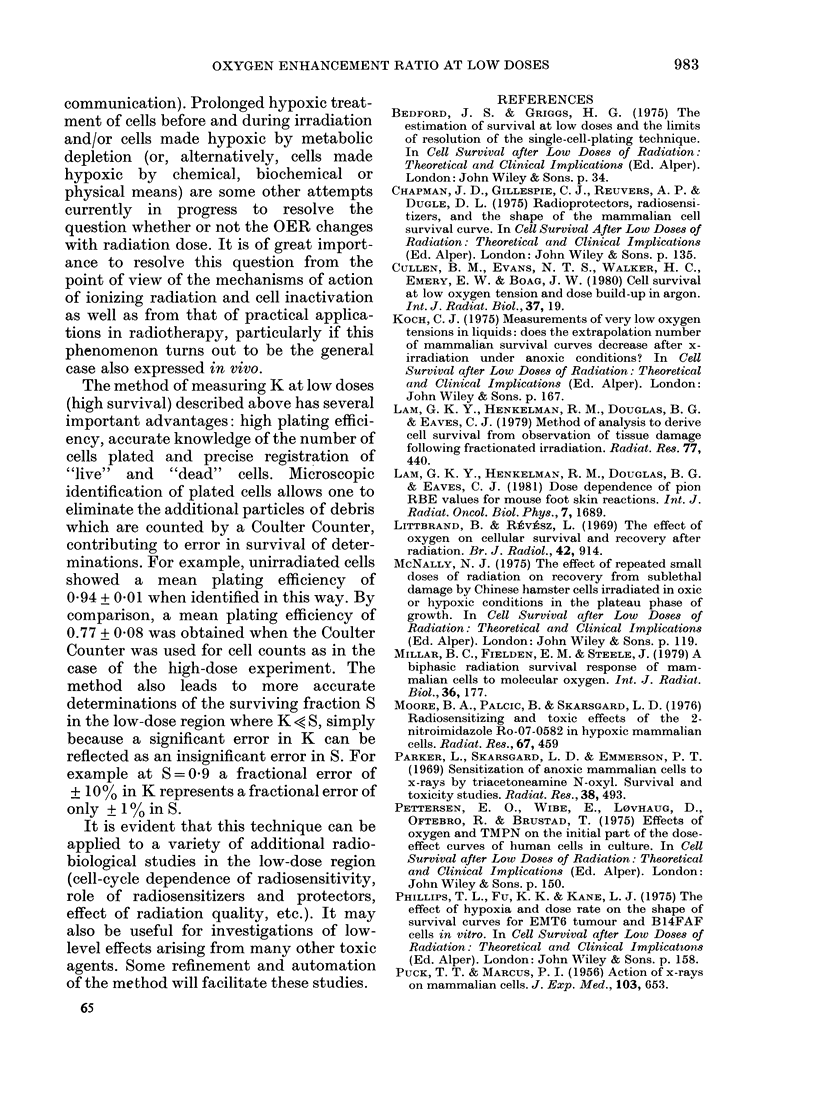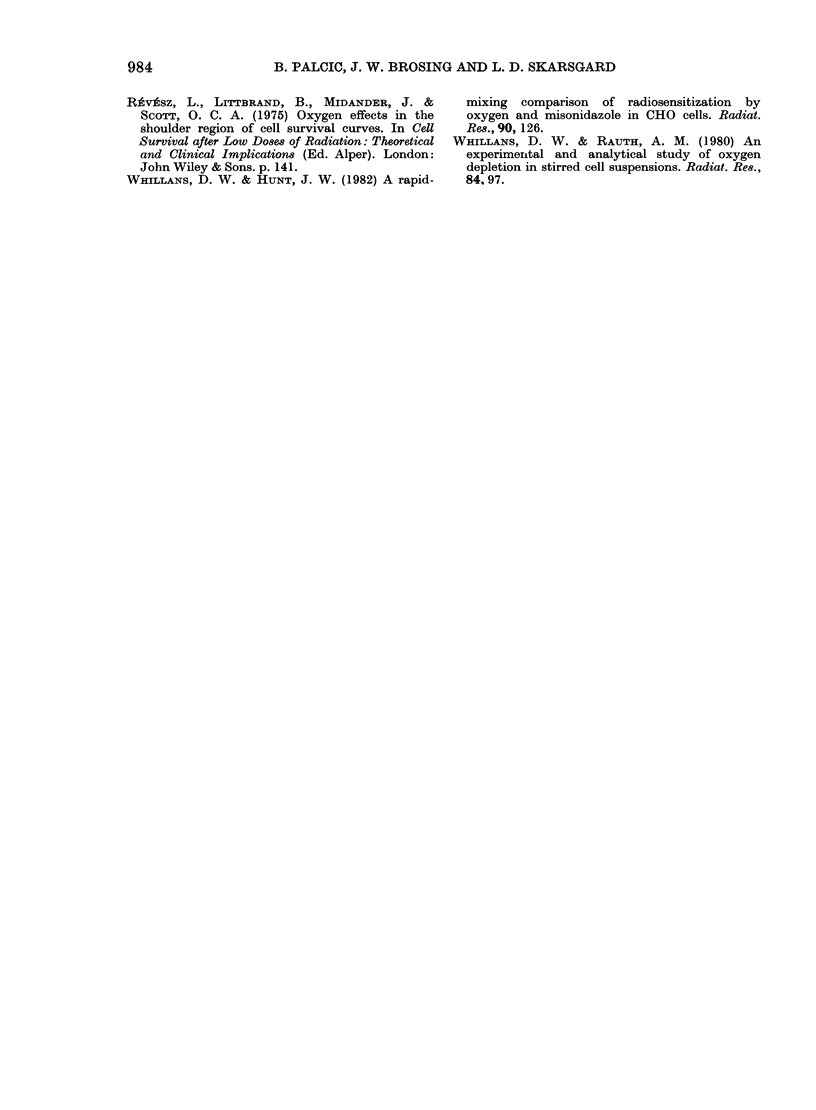# Survival measurements at low doses: oxygen enhancement ratio.

**DOI:** 10.1038/bjc.1982.312

**Published:** 1982-12

**Authors:** B. Palcic, J. W. Brosing, L. D. Skarsgard


*Br. J. Cancer* (1982) **46,** 980

***Short Communication***

### SURVIVAL MEASUREMENTS AT LOW DOSES: OXYGEN ENHANCEMENT RATIO

B. PALCIC, J. W. BROSING AND L. D. SKARSGARD

*From the B.C. Cancer Research Centre, Medical Biophysics Unit,* 601 *West* 10*th Avenue, Vancouver, B.C.* V5Z 1*L*3, *Canada*

THE INACTIVATION of cells by ionizing radiation is usually described by determining the proportion of the population which survives a given radiation exposure. Cells grown *in vitro* are irradiated, counted and plated into Petri dishes, and then incubated at 37°C under growth conditions. After incubation for a prescribed time, the fraction of cells capable of forming a colony of 50 or more cells is determined; these cells are defined as survivors and the logarithm of the fraction of surviving cells, log S, is plotted as a function of dose (Puck & Marcus, 1956).

Measurements in the low-dose region are limited because one is faced with measuring the surviving fraction in a population of predominantly surviving cells. Even when the survival experiment is performed with the utmost care, it is not possible to determine S with precision greater than ~10%. In a log S *vs* D plot, the absolute errors in log S are of the same magnitude throughout the dose range. Thus at low doses (S between 1·0 and 0·5), such uncertainties do not permit determinations of radiobiological parameters (*e.g.* the oxygen enhancement ratio (OER), relative biological effectiveness (RBE) of different radiation modalities, cell-cycle dependence, effects of radiosensitizers and protectors) with sufficient precision to ascertain whether or not these parameters change at low dose as compared to higher doses. The uncertainties in such survival measurements arise primarily from uncertainties in the number of cells plated on the first day. Errors in the counting of cells in suspension, multiple dilutions before plating and the plating itself are all contributory factors.

We have developed a method whereby survival of cells is measured with significantly <10% error. This method is particularly applicable at surviving fractions between 1·0 and 0·5 which corresponds to dose levels between 0 and 3 Gy.

We followed the fate of single cells, plated and identified on Day 1, through the incubation period of 7 days using an inverted microscope. By determining the number of cells which on the 7th day produced a colony of ⩾50 cells, we were able to establish the exact number of surviving cells (S) as well as the number of killed cells (K).

The method of looking at individual cells has been proposed before (Bedford & Griggs, 1975); however, the importance of measuring K (as well as S) was not recognized.

We have used this technique to study the effect of oxygen at high and low doses on survival. Fig. 1 shows the result of a typical survival experiment using Chinese hamster ovary (CHO) cells. An asynchronous population of cells was grown in a spinner flask (Moore *et al.*, 1976). Cells were made hypoxic by flowing purified nitrogen ($N_2$, less than 5 ppm of $O_2$ present) over stirred cell suspensions for at least 45 min before the start of irradiation as well as during irradiation. Cells were kept at 0°C in special glass irradiation vessels

(Parker *et al.*, 1969) in growth medium in a total volume of < 20 ml; cell concentration was kept at $2 \times 10^5$ cells/ml. Aerobic cells were treated identically except that the flow of $N_2$ was not employed. Cells were plated into 5cm plastic Petri dishes (Falcon, with or without an etched grid) and 96-well plastic microtest plates (Nunc, Polystyrene). For high-dose assays, such as presented in Fig. 1, cells were plated so dish with an etched grid or they were plated at an average density of 1 cell/well. Cells were then incubated at 37°C for 7 days. By noting the position of each cell on the day of plating, we could follow the fate of each cell for the incubation period. The use of microtest plates proved to be advantageous in several respects and did not alter the radiation response.

FIG. 1.—Exponentially growing CHO cells were irradiated in dilute suspensions at 0°C under aerobic ($O_2$) or hypoxic ($N_2$) conditions ($2 \times 10^5$ cells/ml in growth medium; X-ray source: Picker, 250 kVp, HVL = 1·7 mm Cu, dose rate 0·3–2 Gy $min^{-1}$). Cells were then diluted, counted and plated into Petri dishes. After a 7-day incubation period, the surviving fraction S was determined. Solid lines through the experimental points represent the least-squares fit to the survival model $S = e^{-\alpha D - \beta D^2}$. The shaded region represents the survival/dose region shown in Fig. 2.

FIG. 2.—Cells were treated as described in Fig. 1 except that after irradiation they were plated into microtest plates (and/or Petri dishes). The growth of individual cells was followed microscopically over a 7-day period, so that the fraction of killed cells, K, as well as the surviving fraction S, was measured. Each point represents a minimum of 8 microtest plates (approximately 100 cells/plate) from at least 4 independent experiments. The criterion for surviving cells is the same as in Fig. 1 (50 cells or more/colony in 7 days). The data were again fitted to the quadratic equation as described in the caption to Fig. 1.

that ~150 colonies emerged after 7 days, irrespective of the conditions of irradiation. The number of cells plated was determined using a Coulter Counter and stained colonies were counted 7 days later.

For the low-dose assay (Fig. 2), approximately 400 cells were plated per 5cm Petri

Within the limits of the experimental error, one finds no difference in the survival values measured by the high-dose assay (standard technique) and the low-dose assay. These measurements were performed at ~0·7 survival level.

It is evident, by comparing Figs 1 and 2, that the separation between $O_2$ and $N_2$ curves is reduced in the low-dose region, indicating a smaller oxygen effect. From the data of Fig. 2, the OER was determined at various doses using an iterative parametric analysis procedure (Lam *et al.*, 1979, 1981) and the results appear in Fig. 3, plotted as a function of the radiation dose delivered in the presence of $O_2$. The data can be adequately represented by a straight line (on a linear dose scale) indicating an OER value at the lowest dose of ~1·5. At the highest dose measured (1·5 Gy in $O_2$) the OER is ~2.

FIG. 3.—Solid circles represent the OER values calculated from the actual data points in Fig. 2. The data points for $O_2$ are transformed using a dose-dependent OER described by the equation: OER = a + bD. The parameters a and b are fitted by an iterative procedure (see text) which minimizes the squared errors in S (least squares fitting) and which also gives the uncertainties associated with each OER value. The error bars represent 1 standard error (63% confidence limit). For comparison, the OER value at higher dose (at S = 0·01) and calculated from the data in Fig. 1, open circle is shown.

The question as to whether or not the oxygen effect is reduced at lower radiation doses (or even non-existent) has been posed several times before. Some reports in the literature suggest that the extrapolation number (n) is reduced for cells irradiated in extreme hypoxia (*e.g.* Littbrand & Révész, 1969) while others would argue that this is not the case (*e.g.* Cullen *et al.*, 1980). Some researchers have presented evidence indicative of a diminished OER at low doses of ionizing radiation in mammalian cells irradiated *in vitro* (Révész *et al.*, 1975; Chapman *et al.*, 1975; McNally, 1975; Pettersen *et al.*, 1975), while others have claimed that the OER is constant throughout the dose range (Phillips *et al.*, 1975; Koch, 1975). All these results were obtained by the standard technique of measuring cell survival.

Our results would support the idea that there is a different OER at low doses, where the survival of cells approaches unity. It has been pointed out (Koch, 1975) that a decreased OER at low doses may be the result of the presence of small amounts of oxygen at low doses in the hypoxic samples, which is then depleted by radiation, yielding an apparent increase of OER at higher doses. At the present time we cannot totally exclude this possibility as the exact $O_2$ concentration in our samples cannot be measured. Nevertheless, the explanation that the observed OER at lower doses is due solely to some traces of oxygen at low doses is unlikely. For CHO cells, one obtains full OER (at high doses) when the oxygen concentration approaches 0·1–0·2 μM in solution (*e.g.* Millar *et al.*, 1979; Whillans & Hunt, 1982). For the observed OER of 1·5 at 0·5 Gy, the oxygen concentration would have to be approximately 3 μM if this were the explanation. Whillans & Rauth (1980) showed that under identical conditions—Type II vessels, gassing purified $N_2$ of less than 9 ppm (in our case < 5 ppm) for over 45 min—the oxygen concentration in solutions is < 0·2 μM. Thus we should attain a full oxygen effect even at these low doses.

Many experiments involving measurements of the exact oxygen levels below concentrations of a few μM will now be performed; the equipment to do these types of measurement will soon be available to us (Koch, 1982 personal communication). Prolonged hypoxic treatment of cells before and during irradiation and/or cells made hypoxic by metabolic depletion (or, alternatively, cells made hypoxic by chemical, biochemical or physical means) are some other attempts currently in progress to resolve the question whether or not the OER changes with radiation dose. It is of great importance to resolve this question from the point of view of the mechanisms of action of ionizing radiation and cell inactivation as well as from that of practical applications in radiotherapy, particularly if this phenomenon turns out to be the general case also expressed *in vivo*.

The method of measuring K at low doses (high survival) described above has several important advantages: high plating efficiency, accurate knowledge of the number of cells plated and precise registration of "live" and "dead" cells. Microscopic identification of plated cells allows one to eliminate the additional particles of debris which are counted by a Coulter Counter, contributing to error in survival of determinations. For example, unirradiated cells showed a mean plating efficiency of $0{\cdot}94 \pm 0{\cdot}01$ when identified in this way. By comparison, a mean plating efficiency of $0.77 \pm 0{\cdot}08$ was obtained when the Coulter Counter was used for cell counts as in the case of the high-dose experiment. The method also leads to more accurate determinations of the surviving fraction S in the low-dose region where $K \ll S$, simply because a significant error in K can be reflected as an insignificant error in S. For example at $S = 0{\cdot}9$ a fractional error of $\pm 10\%$ in K represents a fractional error of only $\pm 1\%$ in S.

It is evident that this technique can be applied to a variety of additional radiobiological studies in the low-dose region (cell-cycle dependence of radiosensitivity, role of radiosensitizers and protectors, effect of radiation quality, etc.). It may also be useful for investigations of low-level effects arising from many other toxic agents. Some refinement and automation of the method will facilitate these studies.

### REFERENCES

Bedford, J. S. & Griggs, H. G. (1975) The estimation of survival at low doses and the limits of resolution of the single-cell-plating technique. In *Cell Survival after Low Doses of Radiation: Theoretical and Clinical Implications* (Ed. Alper). London: John Wiley & Sons. p. 34.

Chapman, J. D., Gillespie, C. J., Reuvers, A. P. & Dugle, D. L. (1975) Radioprotectors, radiosensitizers, and the shape of the mammalian cell survival curve. In *Cell Survival After Low Doses of Radiation: Theoretical and Clinical Implications* (Ed. Alper). London: John Wiley & Sons. p. 135.

Cullen, B. M., Evans, N. T. S., Walker, H. C., Emery, E. W. & Boag, J. W. (1980) Cell survival at low oxygen tension and dose build-up in argon. *Int. J. Radiat. Biol.*, **37**, 19.

Koch, C. J. (1975) Measurements of very low oxygen tensions in liquids: does the extrapolation number of mammalian survival curves decrease after x-irradiation under anoxic conditions? In *Cell Survival after Low Doses of Radiation: Theoretical and Clinical Implications* (Ed. Alper). London: John Wiley & Sons. p. 167.

Lam, G. K. Y., Henkelman, R. M., Douglas, B. G. & Eaves, C. J. (1979) Method of analysis to derive cell survival from observation of tissue damage following fractionated irradiation. *Radiat. Res.* **77**, 440.

Lam, G. K. Y., Henkelman, R. M., Douglas, B. G. & Eaves, C. J. (1981) Dose dependence of pion RBE values for mouse foot skin reactions. *Int. J. Radiat. Oncol. Biol. Phys.*, **7**, 1689.

Littbrand, B. & Révész, L. (1969) The effect of oxygen on cellular survival and recovery after radiation. *Br. J. Radiol.*, **42**, 914.

McNally, N. J. (1975) The effect of repeated small doses of radiation on recovery from sublethal damage by Chinese hamster cells irradiated in oxic or hypoxic conditions in the plateau phase of growth. In *Cell Survival after Low Doses of Radiation: Theoretical and Clinical Implications* (Ed. Alper). London: John Wiley & Sons. p. 119.

Millar, B. C., Fielden, E. M. & Steele, J. (1979) A biphasic radiation survival response of mammalian cells to molecular oxygen. *Int. J. Radiat. Biol.*, **36**, 177.

Moore, B. A., Palcic, B. & Skarsgard, L. D. (1976) Radiosensitizing and toxic effects of the 2-nitroimidazole Ro-07-0582 in hypoxic mammalian cells. *Radiat. Res.*, **67**, 459

Parker, L., Skarsgard, L. D. & Emmerson, P. T. (1969) Sensitization of anoxic mammalian cells to x-rays by triacetoneamine N-oxyl. Survival and toxicity studies. *Radiat. Res.*, **38**, 493.

Pettersen, E. O., Wibe, E., Løvhaug, D., Oftebro, R. & Brustad, T. (1975) Effects of oxygen and TMPN on the initial part of the dose-effect curves of human cells in culture. In *Cell Survival after Low Doses of Radiation: Theoretical and Clinical Implications* (Ed. Alper). London: John Wiley & Sons. p. 150.

Phillips, T. L., Fu, K. K. & Kane, L. J. (1975) The effect of hypoxia and dose rate on the shape of survival curves for EMT6 tumour and B14FAF cells *in vitro*. In *Cell Survival after Low Doses of Radiation: Theoretical and Clinical Implications* (Ed. Alper). London: John Wiley & Sons. p. 158.

Puck, T. T. & Marcus, P. I. (1956) Action of x-rays on mammalian cells. *J. Exp. Med.*, **103**, 653.

Révész, L., Littbrand, B., Midander, J. & Scott, O. C. A. (1975) Oxygen effects in the shoulder region of cell survival curves. In *Cell Survival after Low Doses of Radiation: Theoretical and Clinical Implications* (Ed. Alper). London: John Wiley & Sons. p. 141.

Whillans, D. W. & Hunt, J. W. (1982) A rapid-mixing comparison of radiosensitization by oxygen and misonidazole in CHO cells. *Radiat. Res.*, **90**, 126.

Whillans, D. W. & Rauth, A. M. (1980) An experimental and analytical study of oxygen depletion in stirred cell suspensions. *Radiat. Res.*, **84**, 97.